# Emerging from an Isolation Cocoon, 2022

**DOI:** 10.3201/eid2807.220488

**Published:** 2022-07

**Authors:** Ron Louie

**Affiliations:** University of Washington, Seattle, Washington, USA

**Keywords:** COVID-19, poetry, isolation, masks, misinformation, pandemics, coronavirus disease, SARS-CoV-2, severe acute respiratory syndrome coronavirus 2, viruses, respiratory infections, zoonoses

“The caterpillar does all the work but the butterfly gets all the publicity.”

—Attributed to George Carlin

The security layers started peeling away, seemingly too soon.

Once constricting, every movement and moment a struggle,

who would guess that the loosening would be so worrying?

Preposterous miracles had manifested themselves, albeit

imperfectly; so one emerges, reviving afresh in the sunlight, imbibing

the unmasked scents, even as the serial-killing fiend remains free.

A *kaleidoscope* of butterflies is what one calls a mass fluttering;

that term could well apply to humans here, self-identifying their

unique variants and varieties of existence, behaviors, and beliefs.

The excess of loss has been unthinkable, and not to be forgotten.

Preventive interventions were knowingly imprecise; lacking their own

protections, from denials, was another hazard for the republic’s health.

Poets have long lyricized ideal truth, but the pandemic taught

how fragile truth can be, the fragile beauty of a glistening bubble,

buffeted almost to bursting by a cacophony of ravenous twittering.

Yet one can now stretch out shimmering wings, so to speak,

with the brash confidence befitting a monarch, fully expecting

to start a new cycle in life, despite the circling shadows overhead.

